# Associations of muscle force, power, cross‐sectional muscle area and bone geometry in older UK men

**DOI:** 10.1002/jcsm.12198

**Published:** 2017-05-04

**Authors:** Ayse Zengin, Stephen R. Pye, Michael J. Cook, Judith E. Adams, Rainer Rawer, Frederick C.W. Wu, Terence W. O'Neill, Kate A. Ward

**Affiliations:** ^1^ Nutrition & Bone Health, Elsie Widdowson Laboratory Medical Research Council Human Nutrition Research Fulbourn Rd CB1 9NL Cambridge UK; ^2^ Arthritis Research UK Centre for Epidemiology, Division of Musculoskeletal and Dermatological Sciences, School of Biological Sciences, Faculty of Biology, Medicine, and Health, Manchester Academic Health Science Centre The University of Manchester Oxford Road Manchester M13 9PT UK; ^3^ Radiology and Manchester Academic Health Science Centre (MAHSC) Manchester Royal Infirmary, Central Manchester University Hospitals NHS Foundation Trust and University of Manchester Oxford Road Manchester M13 9WL UK; ^4^ Novotec Medical GmbH Durlacher Str. 35 Pforzheim 75172 Germany; ^5^ Andrology Research Unit, Centre for Endocrinology and Diabetes, Faculty of Medical and Human Sciences, Manchester Academic Health Science Centre (MAHSC) The University of Manchester, Central Manchester University Hospitals NHS Foundation Trust Old St Mary's Building, Hathersage Road Manchester M13 9WL United Kingdom; ^6^ NIHR Manchester Musculoskeletal Biomedical Research Unit Central Manchester University Hospitals NHS Foundation Trust, Manchester Academic Health Science Centre Grafton Street Manchester M13 9WL UK; ^7^ Department of Rheumatology Salford Royal NHS Foundation Trust Stott Lane Salford M6 8HD UK; ^8^ Medical Research Council Lifecourse Epidemiology Unit University of Southampton Tremona Road Southampton SO16 6YD UK

**Keywords:** Jump force, Jump power, Bone geometry, Sarcopenia, Osteoporosis

## Abstract

**Background:**

Ageing is associated with sarcopenia, osteoporosis, and increased fall risk, all of which contribute to increased fracture risk. Mechanically, bone strength adapts in response to forces created by muscle contractions. Adaptations can be through changes in bone size, geometry, and bending strength. Muscle mass is often used as a surrogate for muscle force; however, force can be increased without changes in muscle mass. Increased fall risk with ageing has been associated with a decline in muscle power—which is a measure of mobility. The aims of this study were as follows: (i) to investigate the relationship between muscle parameters in the upper and lower limbs with age in UK men and the influence of ethnicity on these relationships; (ii) to examine the relationships between jump force/grip strength/cross‐sectional muscle area (CSMA) with bone outcomes at the radius and tibia.

**Methods:**

White European, Black Afro‐Caribbean, and South Asian men aged 40–79 years were recruited from Manchester, UK. Cortical bone mineral content, cross‐sectional area, cortical area, cross‐sectional moment of inertia, and CSMA were measured at the diaphysis of the radius and tibia using peripheral quantitative computed tomography. Lower limb jump force and power were measured from a single two‐legged jump performed on a ground‐reaction force platform. Grip strength was measured using a dynamometer. Associations between muscle and bone outcomes was determined using linear regression with adjustments for age, height, weight, and ethnicity.

**Results:**

Three hundred and one men were recruited. Jump force was negatively associated with age; for every 10 year increase in age, there was a 4% reduction in jump force (*P* < 0.0001). There was a significant age–ethnicity interaction for jump power (*P* = 0.039); after adjustments, this was attenuated (*P* = 0.088). For every 10 year increase in age, grip strength decreased by 11%. Jump force was positively associated with tibial bone outcomes: a 1 standard deviation greater jump force was associated with significantly higher cortical bone mineral content 3.1%, cross‐sectional area 4.2%, cortical area 3.4%, and cross‐sectional moment of inertia 6.8% (all *P* < 0.001). Cross‐sectional muscle area of the lower leg was not associated with tibial bone outcomes. Both grip strength and CSMA of the arm were positively associated, to a similar extent, with radius diaphyseal bone outcomes.

**Conclusions:**

Jump force and power are negatively associated with age in UK men. In the lower limb, the measurement of jump force is more strongly related to bone outcomes than CSMA. It is important to consider jump force and power when understanding the aetiology of bone loss and mobility in ageing men.

## Introduction

Ageing is associated with sarcopenia, loss of muscle strength, osteoporosis, and increased fall risk, all of which contribute to an increased risk of fracture.^1^, ^2^ Muscle strength not only includes the amount of muscle (mass) but also anatomy (type and distribution of muscle fibres), force (the product of mass and acceleration), and power.^3^ Muscle mass, measured from dual energy X‐ray absorptiometry scans or cross‐sectional muscle area (CSMA) derived from peripheral quantitative computed tomography (pQCT) scans are often used as a surrogate for muscle force. Data from population studies involving older men and women have shown that the decline in muscle force is significantly greater than the decline in muscle mass,^4^, ^5^ indicating that the force generated by muscle contractions is not proportional to muscle mass.

Mobility, the ability to move without assistance, locomotion, and balance, are dependent on muscle power,^6^, ^7^, ^8^ which is the product of force and velocity, reflecting the ability of how fast muscles can produce force and so maintain stance or motion. The increased risk of falls with ageing has been associated with a decline in muscle power.^9^, ^10^ A new approach to the measurement of muscle force and power is jumping mechanography, which enables the real‐time recording of force, velocity, and power in the leg from a ‘usual’ daily task and so may be more useful than the traditional tests.^11^ Jumping mechanography has been validated as a reproducible tool,^12^, ^13^, ^14^ enabling site‐specific assessment corresponding to loading, falls, and fracture in the elderly. A study has shown that there was a stronger association in the decline in jump power with age than for chair‐rising power^15^ and suggests that jumping mechanography may be more sensitive in detecting the effects of ageing on muscle power compared with the chair‐rise test.

A decline in muscle force has been associated with fracture risk. Data from a recent longitudinal study examining quadriceps isometric muscle force in individuals aged 60 years and above have shown that for each 1 standard deviation (SD) lower muscle force, there was an increased risk in sustaining a fracture (any) by 27% in women and 46% in men.^16^ Another traditional measure, grip strength, has been shown to be a good predictor of morbidity, mortality, and bone health.^17^, ^18^ A study in men aged 60 years and over has demonstrated that men in the lowest grip strength quartile had lower total volumetric bone mineral density (vBMD), cortical area (Ct.area), and cortical thickness (measured with pQCT) at the radius vs. men in the highest quartile.^19^ Jump force assessed by jumping mechanography has been shown to be positively correlated with cortical bone area and cortical thickness in men aged 25–45 years,^20^ suggesting that the force generated by muscle contractions has a positive effect on cortical bone parameters. We recently reported differences in the relationships between bone and age between different ethnic groups,^21^ and it is known that fracture risk varies widely between these groups.^22^ Differences in body habitus (muscle mass, fat mass, weight, and height) have been shown to be a major contributor to the reported differences in fracture risk^3^, ^23^; the contribution of muscle force and power to these ethnic differences is unknown.

Therefore, the aims of this study were to investigate the relationship between muscle parameters in the upper and lower limbs with age in men from the UK and to examine whether there were any ethnic differences in these relationships. We then investigated the relationships between jump force/CSMA and bone outcomes at the diaphysis of the radius and tibia.

## Study design

### Subjects

White European men aged 40 years and over were recruited from primary care registers in Manchester (UK) for participation in the European Male Aging Study.^24^ Briefly, participants were invited by letter of invitation to attend a local clinic for assessment, including an interviewer‐assisted questionnaire. They were subsequently invited to attend a follow‐up assessment approximately 4.3 years later at which time they had pQCT measurements at the radius and tibia and jumping mechanography. At the time of the follow‐up assessment, an additional sample of men aged 40 years and over of Black Afro‐Caribbean descent and South Asian men of Pakistani, Bangladeshi, or Indian descent was invited to attend for the same suite of assessments.^21^ Recruitment for these ethnic groups was through a combination of approaches including advertising in community centres and through local media. There were no specific exclusion criteria apart from participants being able to provide written, informed consent for the main European Male Aging Study. However, for this add‐on study, physical capability was assessed on attendance using the stair climb test in community‐dwelling men. Ethical approval for the study was obtained from the North West Multi Centre Ethical Research Committee in Manchester and has been performed in accordance with the ethical standards laid down in the 1964 Declaration of Helsinki and its later amendments. All participants provided written informed consent. Height was measured using a stadiometer (Leicester Height Measure, SECA UK Ltd), and body weight was measured using an electronic scale (SECA UK Ltd). Body mass index was calculated as weight in kilograms (kg) divided by the square of height (m).

### Peripheral quantitative computed tomography

Peripheral quantitative computed tomography measurements were made at the radius and tibia using a Stratec XCT‐2000 scanner (Stratec, Pforzheim, Germany). All measurements were made in the non‐dominant limb. Measurements were taken at 50% (radius) and 38% and 66% (tibia) of the limb length. Forearm length was defined as the distance from the styloid process of the ulna to the olecranon. Tibia length was defined as the distance from the most distal edge of the medial malleolus to the medial intercondylar eminence of the tibia at the knee and was measured using a segmometer. The scan sites were determined using a CT planar scout view of the distal radius or tibia, and the reference lines were placed to bisect the medial border of the distal radial joint surface and being parallel to the distal joint surface of the tibia. A number of participants had legs which were too large to position ideally in the pQCT scanner gantry, which resulted in scanning at the 50% site instead of the 66% site. Cortical bone mineral content (BMC) (mg/mm), cross‐sectional area (CSA) (mm^2^), Ct.area (mm^2^), and cross‐sectional moment of inertia (CSMI) (mm^4^)—as a measure of the bone's resistance to buckling—were measured. All scans at the 50% radius and 38% tibia were analysed using separation mode 1, threshold = 710 mg/cm^3^ for cortical bone outcomes. For CSMA, scans were analysed using contour mode 3, threshold = 40 mg/cm^3^ and peel mode 1. Where significant motion artefact was detected, scans were excluded. The short term precision of two repeat radius measurements with repositioning in adults was (*n* = 22): Ct.area 2.4%, CSMA 3.7%. Manufacturer's standard quality assurance procedures were followed. All pQCT images were reported by an experienced musculoskeletal radiologist using the manufacturer's software version 6.20, as previously described.^25^

### Jumping mechanography

To assess lower limb force and power, individuals were asked to perform the single two‐legged jump on the Leonardo Ground Reaction Force Platform (Leonardo software version 4.2; Novotec Medical GmbH) as described previously.^26^ Briefly, individuals were asked to jump, bending their knees to jump as high as possible. The jump test was repeated three times, and the jump with the highest jumping height was used for analysis. Jump power (kW) and jump force (kN) were measured and were also normalized to body weight and named relative jump power (W/kg) and relative jump force (N/kg) by the software. The Esslinger fitness index (%) is calculated by dividing the measured value of relative jump power of a participant by the mean of a sex and age‐matched reference.^15^, ^27^ The intra and inter‐rater reliability of jumping mechanography has been reported^13^, ^14^ with a CV of 0.3–0.6% in 10 healthy adults.

### Grip strength

Grip strength (kg) was measured using a dynamometer (Jamar Hand Dynamometer, IL, USA). The individual was seated in an upright position and with the arm of the measured hand unsupported and parallel to the body. For each individual, we allowed one test trial and then took three test measurements and used the highest measurement in our analysis.

### Statistical analysis

Descriptive statistics were used to describe the subject characteristics. Between‐group differences in the subject characteristics were tested by one‐way analysis of variance. We then explored the relationship between muscle parameters (grip strength, force, power, CSMA; dependent variable) with age (independent variable) using linear regression with adjustments for weight and height. To test if these relationships were different between ethnic groups, we included an ethnicity*age term; if significant, the *P*‐value from the relevant pairwise comparison was reported, and otherwise the interaction term was removed. Muscle parameters were log transformed to normalize distributions and to allow expression of the results as percent change per unit (10 year) increase in age with the results expressed as beta coefficients with 95% confidence intervals.^28^ The proportion of variance explained by the linear regression models was calculated using the *R* ^2^ statistic. To facilitate the interpretation of the results, grip strength, jump force, jump power, radius, and tibia CSMA values were transformed into *z*‐scores (per SD) (*Figure* 2).

Second, we used linear regression to investigate the relationship between bone outcomes (dependent variable) and either grip strength, force, or CSMA (independent variable), with adjustments for ethnicity, age, weight, and height; we tested also for a grip strength/force/CSMA*ethnicity interaction using a Wald test. Interaction terms in both models were removed if non‐significant. Bone outcomes were log transformed to normalize distributions as described previously.^28^ Values are presented as beta coefficients expressed as a percent change for every 1SD change in grip strength/force/CSMA with 95% confidence intervals. There were no significant differences in using either absolute jump force or relative jump force in the regressions; data presented are from the analyses using absolute jump force. A *z*‐test was used to test whether or not there were differences in using grip strength/force compared with CSMA when investigating the associations with bone outcomes at the radius and tibia. All analyses were performed in Stata, Version 14.0 (StataCorp, College Station, TX, USA), and we considered results statistically significant at *P* < 0.05.

## Results

In total, 301 participants—201 White, 43 Black Afro‐Caribbean, and 57 South Asian men—were studied. White men were slightly older than Black and South Asian men. South Asian men were slightly shorter compared with White men; Black men were not different to neither White nor South Asian men; there were no ethnic differences in either weight or body mass index (*Table* 1). Unless stated, there were no significant ethnic interactions.

**Table 1:** Descriptive characteristics

	Mean ± SD (*n* = 301)
*General characteristics*	
Age (year)	62.5 ± 11.0
Weight (kg)	83.8 ± 12.0
Height (cm)	172.7 ± 6.9
BMI (kg/m^2^)	28.1 ± 3.6
Radius length (cm)	28.1 ± 1.5
Tibia length (cm)	40.2 ± 2.3
*50% radius*	
Ct. vBMD (mg/cm^3^)	1215.4 ± 31.0
Ct.BMC (mg/mm)	123.1 ± 18.5
CSA (mm^2^)	149.2 ± 20.9
Ct. area (mm^2^)	107.1 ± 15.0
CSMI (mm^4^)	1648 ± 433
CSMA (mm^2^)	3631 ± 689
*38% tibia*	
Ct. vBMD (mg/cm^3^)	1188.6 ± 40.8
Ct.BMC (mg/mm)	398.0 ± 56.4
CSA (mm^2^)	478.4 ± 55.9
Ct. area (mm^2^)	346.1 ± 45.5
CSMI (mm^4^)	16966 ± 3897
*66% tibia*	
CSMA (mm^2^)	7216 ± 1066
*Muscle parameters*	
Grip strength (kg)	37.3 ± 10.0
Jump force (kN)	1.93 ± 0.37
Jump power (kW)	2.77 ± 0.76
Relative jump force (N/kg)	22.9 ± 3.4
Relative jump power (W/kg)	32.4 ± 8.2
EFI (%)	82.7 ± 16.2

All values are mean ± SD. BMI, body mass index; CSA, cross‐sectional area; CSMA, cross‐sectional muscle area; CSMI, cross‐sectional moment of inertia; Ct, cortical; EFI, Esslinger fitness index; kg, kilograms; kN, kilo Newtons; kW, kilo Watts; BMC, bone mineral content; vBMD, volumetric bone mineral density.

### Relationship between muscle parameters and age

**Figure 1:**
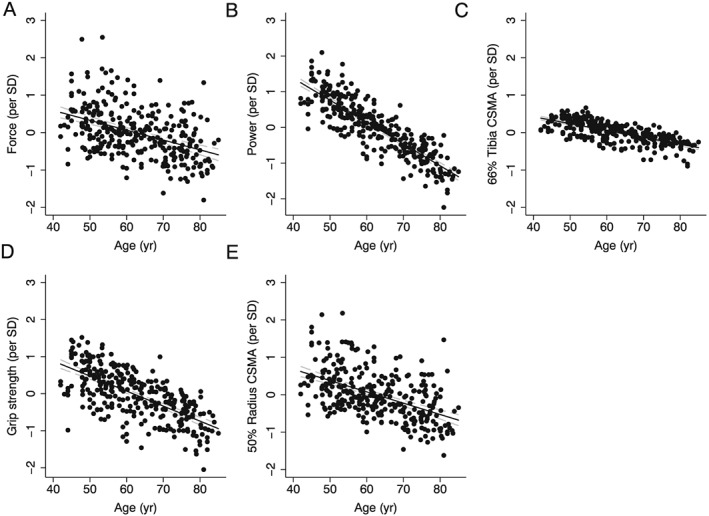
The relationship between age and *(A)* jump force *(B)* jump power *(C)* CSMA at the 66% tibia *(D)* grip strength and *(E)* CSMA at the 50% radius in men. Adjustments were made for ethnicity, weight, and height.

*Lower limb:* For every 10 year increase in age, there was a 4% decrease in jump force following adjustments for weight, height, and ethnicity (*Figure* 1A, *P* < 0.0001). There was a negative association between jump power and age (*Figure* 1B), and in the unadjusted analyses, there was a significant age–ethnicity interaction term (*P* = 0.039); following adjustments for body size, this interaction became non‐significant (*P* = 0.088). For every 10 year increase in age, there was a 4% reduction of CSMA in body size adjusted models (*Figure* 1C, *P* < 0.0001).

*Upper limb:* For every 10 year increase in age, there was an 11% reduction in grip strength in body size adjusted models (*Figure* 1D). Cross‐sectional muscle area of the arm was negatively associated with age (*Figure* 1E); for every 10 year increase in age, there was a 5% decrease in CSMA in body size adjusted models (*P* < 0.0001).

### Muscle and bone relationship at the tibia

**Figure 2:**
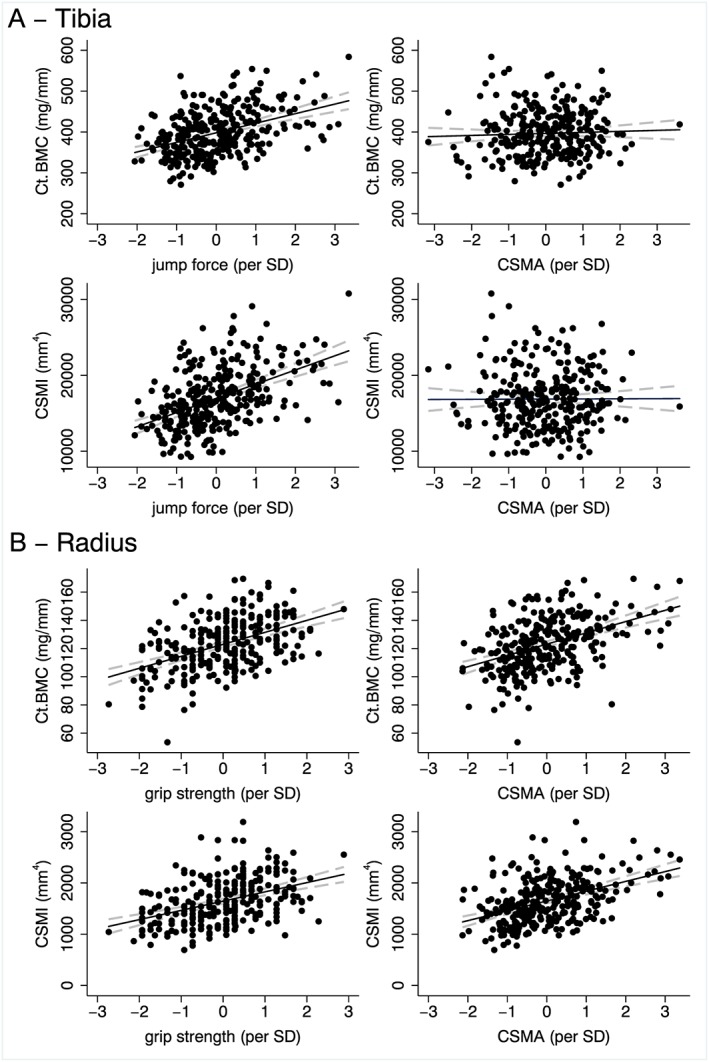
Scatter plot and linear regression line (solid black line) with 95% confidence intervals (grey dashed line) representing the relationship between *(A)* tibial outcomes at the 38% site and jump force/CSMA (per SD); *(B)* radius outcomes at the 50% site and grip strength/CSMA (per SD). Ct.BMC (cortical bone mineral content); CSMI (cross‐sectional moment of inertia); CSMA (cross‐sectional muscle area).

There were positive relationships between force *z*‐score and bone outcomes at the 38% tibia (*Table* 2). For every 1SD increase in force, there was a 3% greater Ct.BMC (*Figure* 2A). The strongest association was between CSMI and force *z*‐score; for every 1SD increase in force, there was a 7% greater CSMI (*Figure* 2A). In contrast, there were no significant associations between CSMA *z*‐score and tibial bone outcomes (*Figure* 2A, *Table* 2). The *z*‐test showed that the use of jump force compared with CSMA better predicted tibial bone measures: Ct.BMC (*P* = 0.02), CSA (*P* < 0.001), Ct.area (*P* = 0.01), and CSMI (*P* < 0.001) following adjustments and *P* < 0.001 at all sites in unadjusted models.

**Table 2:** The relationship between force and CSMA with bone outcomes at the tibia

	Force (per SD)			CSMA (per SD)		
	*Β* (%)	*R* ^2^	*P* value	*Β* (%)	*R* ^2^	*P* value
38% tibia						
Ct.BMC (mg/mm)	3.1 (1.3, 4.9)	0.31	0.001	0.2 (−1.2, 1.7)	0.27	0.770
CSA (mm^2^)	4.2 (2.4, 6.0)	0.33	<0.0001	0.003 (−1.5, 1.5)	0.28	0.997
Ct. area (mm^2^)	3.4 (1.8, 5.0)	0.34	<0.0001	0.4 (−1.0, 1.7)	0.29	0.588
CSMI (mm^4^)	6.8 (4.0, 9.5)	0.40	<0.0001	−0.02 (−2.3, 2.2)	0.34	0.984

All values are beta coefficients expressed as a percent change for every 1SD change in force/CSMA with 95% confidence intervals. Adjustments were made for ethnicity, age (year), weight (kg), and height (cm); bold indicates *P* < 0.05. CSA, cross‐sectional area; CSMA, cross‐sectional muscle area; CSMI, cross‐sectional moment of inertia; Ct, cortical; BMC, bone mineral content.

### Muscle and bone relationship at the radius

There were positive relationships between grip strength *z*‐score and cortical BMC, CSA and Ct.area, at the 50% radius (*Table* 3), which together contributed to greater CSMI (*Figure* 2B); similar relationships were found with CSMA. For every 1SD increase in CSMA, there was a 12% increase in CSMI (*Table* 3). The *z*‐test showed that either CSMA or grip strength similarly predicted bone outcomes.

**Table 3:** The relationship between grip strength and CSMA with bone outcomes at the radius

	Grip strength (per SD)			CSMA (per SD)		
	*Β* (%)	*R* ^2^	*P* value	*Β* (%)	*R* ^2^	*P* value
50% radius						
Ct.BMC (mg/mm)	5.3 (3.4, 7.3)	0.40	<0.0001	3.7 (1.8, 5.6)	0.37	<0.0001
CSA (mm^2^)	5.6 (3.7, 7.6)	0.27	<0.0001	6.6 (4.8, 8.4)	0.29	<0.0001
Ct. area (mm^2^)	5.7 (4.0, 7.7)	0.43	<0.0001	4.7 (3.0, 6.4)	0.41	<0.0001
CSMI (mm^4^)	11.3 (7.8, 14.9)	0.33	<0.0001	12.2 (8.9, 15.6)	0.34	<0.0001

All values are beta coefficients expressed as a percent change for every 1SD change in grip strength/CSMA with 95% confidence intervals. Adjustments were made for ethnicity, age (yr), weight (kg) and height (cm); bold indicates *P* < 0.05. CSA, cross‐sectional area; CSMA, cross‐sectional muscle area; CSMI, cross‐sectional moment of inertia; Ct, cortical; BMC, bone mineral content.

## Discussion

Using jumping mechanography, which is a novel method to directly assess lower limb force and power, we have shown negative relationships between jump force and power with age in older (aged ≥40 years) community‐dwelling men. Jump force, a measure of loading, was positively associated with the amount of mineral, bone size, and strength at the tibia, although CSMA of the lower leg was not. In contrast, there were similar associations between grip strength and CSMA of the arm with diaphyseal bone outcomes at the radius. Taken together, the data from our study show the importance to consider measurement of jump force and power when understanding lower limb bone health and mobility in ageing men.

Our findings are consistent with the mechanostat theory that the peak mechanical loads primarily come from force generated by skeletal muscle contractions.^29^ A study in healthy men aged 50–87 years showed that a higher lifetime history of weight‐bearing exercises was associated with mid‐femur bone size, cortical area, and resistance to torsion.^30^ In our study, we found that jump force most strongly influences CSMI at the tibial diaphysis. Cross‐sectional moment of inertia is a measure of bone bending strength adapting in response to bending loads on the bone.^31^ At the diaphysis of the tibia, bending, rather than compressive loads as at distal sites, are the predominant drivers of change in bone geometry to maintain strength.^31^, ^32^ Cross‐sectional moment of inertia was reported to be a better predictor of bone fracture than measurements of CSA.^33^ Whilst we do not have fracture data in this study, the positive associations between jump force and CSMI are consistent with studies that have shown an increased risk of fracture with a decline in muscle force.^16^

Our findings show negative associations between jump force, power, and CSMA with age in older (aged ≥40 years) community‐dwelling men residing in Manchester, UK. However, there was a three‐fold greater change in jump power with age compared with CSMA. These data show that the decline with age in jump power is far greater than the decline with age in CSMA, which is likely to have a greater impact on mobility and locomotion than loss of muscle mass among older adults. A study demonstrated that older people (~72 years of age) with similar muscle mass to that of younger people (~22 years of age) covered a shorter distance in the 6 min walk test.^34^ Studies have shown that older people who fall have less muscle power in the lower limbs compared with their non‐faller counterparts,^10^, ^35^ suggesting that muscle power is a determinant of fall risk.

In this study, we show strong positive associations between grip strength and structural parameters at the radius diaphysis. Our findings are consistent with the Osteoporotic Fractures in Men Study, which has shown that men in the lowest quartile of grip strength had smaller bones, lower cortical area, and stress strain index at the diaphysis of the radius.^36^ Similarly, in the Hertfordshire Cohort Study, positive associations in men aged 70 years and over between grip strength and diaphyseal periosteal circumference, stress strain index, and cortical bone area were reported.^37^ Consistent with previous studies, we also show a negative relationship between grip strength and age. Combined data from 12 general population studies conducted in the UK have shown that grip strength increases to a peak in early adult life followed by a period of maintenance and then declines with increasing age.^38^ A longitudinal study in men and women aged 85 years in Leiden (The Netherlands) showed that mortality increased among participants in the tertile with the highest relative loss of handgrip strength over four years.^18^

Our findings highlight important differences between the assessment of muscle–bone relationships in the upper and lower limbs. In prospective studies, it will be important to consider these differences. Grip strength and CSMA were similarly associated with outcomes at the radius diaphysis. In contrast, diaphyseal tibial outcomes were significantly predicted by jump force and not CSMA. Grip strength involves movement of the thumb, fingers, and wrist and use of muscles of the forearm—most of which have their origin at the humerus and insertion into metacarpals. Anatomically, these muscles are not exerting loads upon the diaphysis of the radius during the grip strength test; muscle forces are more influential near muscle insertions due to high tensile stresses at the junction where the tendon attaches to the periosteal surface of the long bone shaft.^39^ Therefore, in a non‐weight bearing site, and non‐elite population, it is perhaps not surprising that grip strength is no better than CSMA in predicting bone outcomes. In contrast at a weight‐bearing site, the tibia, where all muscles in the lower leg will at some point load the tibia during daily movements and jumping, we found associations with jump force and bone, while there were no associations with CSMA and bone. This is because bending loads are not only caused by regional muscles attached to the bone studied but also by muscles that actuate neighbouring and distant segments^32^; for instance, at the tibia, the knee extensors exert bending loads, which increase during the flexion of the knee—a movement occurring during a jump. In addition, the tibia is also loaded through the thigh muscles during standing up and jumping from deep squats.^32^ Notably, the quadriceps femoris exerts bending loads on the tibia and the knee joint through the patellar tendon, especially when the knees are flexed, during a jump.^40^ Together, these findings suggest that the muscles used during a jump exert forces and thereby bending loads on the tibia are similar to those it experiences on a daily basis, indicating that the force measured from a jump provides an accurate estimation of the loads exerted onto the tibia and why tibial CSMA does not predict tibial diaphysis bone outcomes.

There are several potential limitations to this study. The observational and cross‐sectional design does not allow us to draw conclusions about causality or within individual change. The use of a single two‐legged jump instead of the multiple one legged hopping to assess jump force may have resulted in an underestimation of peak forces; multiple one legged hopping yields higher peak voluntary forefoot ground reaction forces.^6^ A number of participants had larger legs in which scanning was performed at the 50% site instead of the 66% site; this affected 12% of individuals. This may have increased the variance in CSMA and bone outcomes and decreased the precision. Among the ethnic minority groups ancestral origins were self‐reported and may have resulted in misclassification; however, we attempted to reduce possible misclassification by requiring that three out of four grandparents were of the same ethnic origin. Differences in recruitment methodologies may have influenced participation in the study, particularly in the Black and South Asian groups. The number of men in the Black and South Asian groups was relatively small, and so caution is required in interpreting the results. We do not have data on falls or fractures and so cannot draw direct associations between these outcomes and muscle power.

We have described the relationship between muscle and bone in both the upper and lower limbs of community‐dwelling ageing UK men. Jump force and grip strength were positively associated with bone outcomes reflecting size, geometry, and bending strength at the diaphysis of the radius and tibia. There were no ethnic differences in muscle–bone relationships. Together these findings highlight the importance of using functional measures of muscle to understand the muscle–bone relationships during ageing. The age‐related decline in muscle force and power may provide a target for intervention to improve both muscle and bone health in older males.

## Conflict of interest

Ayse Zengin, Stephen R Pye, Michael J Cook, Judith E Adams, Frederick CW Wu, Terence W O'Neill, and Kate A Ward declare that they have no conflict of interest. Rainer Rawer is an employee of Novotec Medical GmBH.
